# Evaluation of a stroke rehabilitation training programme for community-based primary healthcare

**DOI:** 10.4102/ajod.v12i0.1137

**Published:** 2023-09-08

**Authors:** Elsje Scheffler, Robert Mash

**Affiliations:** 1 Division of Family Medicine and Primary Care, Faculty of Medicine and Health Sciences, Stellenbosch University, Cape Town, South Africa; 2 Centre for Disability and Rehabilitation Studies, Faculty of Medicine and Health Sciences, Stellenbosch University, Cape Town, South Africa

**Keywords:** primary healthcare, community care, stroke rehabilitation, community health workers, low resourced, low- and middle-income countries

## Abstract

**Background:**

Family caregiver training is an integral part of stroke rehabilitation programmes and is associated with improved caregiver and stroke survivor outcomes. In the Cape Winelands District, a low-resourced rural community-based setting in South Africa, stroke survivors and family caregivers mostly rely on assistance from community health workers (CHWs), despite their lack of stroke-specific rehabilitation training.

**Objectives:**

To evaluate the implementation and immediate effects of a bespoke, 16 session, 21 h stroke rehabilitation training programme for CHWs to better support family caregivers.

**Methods:**

Two cooperative inquiry groups participated in participatory action research to design and develop the programme. This article reports on the implementation of this programme. Inquiry group members directly observed the training, obtained written and verbal feedback, interviewed CHWs and observed them in the community. Consensus on their learning was achieved after reflection on their experience and observations.

**Results:**

Learning of the cooperative inquiry groups was categorised into the effect on community-based care, the training programme’s design and development, how training was delivered and implications for service delivery. Community health workers empowered caregivers and stroke survivors and enabled access to care, continuity, coordination and person-centredness. The need for experiential learning and a spiral curriculum was confirmed. Therapists needed a different set of skills to deliver training. A systems approach and effective leadership were needed to enable community health workers to use their new skills.

**Conclusion:**

The stroke rehabilitation training programme demonstrated potential for integration into service delivery and equipping CHWs to support family caregivers and stroke survivors. Further evaluation of the programme’s effectiveness and scale-up is needed.

**Contribution:**

Evidence of an intervention to train CHWs to support stroke survivors and family caregivers.

## Introduction

Many stroke survivors require ongoing assistance from family members for participation and integration into daily activities, as well as general and rehabilitation health management. Family caregiver training is therefore an essential element of global stroke rehabilitation programmes (Kamalakannan et al. 2016; Langhorne et al. 2018; Pandian et al. 2016; Yan et al. 2016a) and stroke guidelines across all levels of stroke care (Bryer et al. 2011; Cameron et al. 2016; Lindsay et al. 2014; Winstein et al. 2016). Caregiver training is associated with improved stroke survivor and caregiver knowledge and skills (Araújo et al. 2018; Bakas et al. 2014; Camak 2015; Pitthayapong et al. 2017; White, Cantu & Trevino 2015), reduced care burden (Araújo et al. 2018; Bakas et al. 2014; Camak 2015), reduced caregiver anxiety and depression and improved mental health (Araújo et al. 2018). For stroke survivors, caregiver training is associated with reduced depression and anxiety, increased patient satisfaction and a decrease in secondary complications and hospital readmissions and may also improve stroke survivor function and quality of life (Bakas et al. 2014; Pitthayapong et al. 2017). Globally, most caregiver interventions are delivered by rehabilitation professionals as part of stroke unit care and multidisciplinary rehabilitation (Bakas et al. 2014; Camak 2015; Dobe, Gustafsson & Walder 2022; Hafsteinsdóttir et al. 2011; Khondowe, Rhoda & Mpofu 2007; Nordin et al. 2014; Pesantes et al. 2017; The ATTEND Collaborative Group 2017).

Where rehabilitation services and numbers of rehabilitation professionals are low, simpler caregiver training programmes led by general nurses have been advocated for (Zhou et al. 2019) and successfully implemented (Deyhoul et al. 2019; Pitthayapong et al. 2017). With community nursing staff often already overburdened, task-shifting of caregiver training to grassroots- or mid-level workers has been advocated as it falls within their scope and role (Bryer 2009; Bryer et al. 2011; Hassan et al. 2012; Miranda et al. 2017; The ATTEND Collaborative Group 2017; Wasserman, De Villiers & Bryer 2009). However, no evidence of stroke rehabilitation task-shifting to these cadres and/or implementation and evaluation of stroke training programmes were found in low-resourced settings. With the global burden of disease from stroke shifting towards low- and middle-income countries (Donkor 2018; Langhorne et al. 2018), these cadres could become a pivotal resource to support stroke survivors and their caregiver in these settings. However, there is limited evidence on community health worker (CHW) rehabilitation-related training and insufficient information on training methods and outcomes (Abdel-All et al. 2017; Nesbit, Gombwa & Ngalande 2015).

South Africa has recorded high and increased rates of stroke mortality and morbidity, particularly in rural communities (Maredza, Bertram & Tollman 2015; Taylor & Ntusi 2019; The SASPI Project Team 2004). However, rehabilitation services are often inaccessible to stroke survivors because of poverty and other contextual barriers, such as inaccessible or unavailable transport and infrequent service delivery, and stroke survivors are largely dependent on family caregivers only (Cawood & Visagie 2016; Eide et al. 2015; Maart & Jelsma 2014; Visagie & Swartz 2016). Public healthcare policy in South Africa emphasises a primary healthcare (PHC) approach (National Department of Health 2020; ed. Van Rensburg 2012). Acute services are delivered at central, regional and district hospitals. Nurse-driven primary care services are delivered at facilities where rehabilitation is provided by a small roving team of multidisciplinary professionals (MDPs) (Naledi, Barron & Schneider 2011). A community-oriented primary care (COPC) approach integrates facility- and community-based services (CBSs) in defined communities with multidisciplinary and intersectoral cooperation (Mash et al. 2019). In the home- and community-based care (HCBC) platform, COPC is delivered by teams of CHWs led by care coordinators, who are professional and/or enrolled nurses (Naledi et al. 2011). Community health worker s are selected from and work as part of ward-based PHC outreach teams in the communities they live. Their scope includes health promotion and disease prevention, referral to health services, adherence support and counselling for those with chronic conditions, minor curative services, home-based care, which includes facilitation of daily living activities and restoring function, as well as providing psychosocial support.

Historically, pre-1994 under the Apartheid era, numerous innovative ad hoc CHW programmes were delivered by non-government organisations, often with a limited focus, for example, on home-based care, pregnant mothers, nutrition and in some cases disability. Ironically, once PHC was established as policy by the new government post-1994, many of the CHW programmes closed for lack of funding (Van Ginneken, Lewin & Berridge 2010). The government only promoted CHWs again in 2010 as part of PHC reengineering and standardised national accredited training only started in 2016, but with limited rehabilitation-related content (South African Qualifications Authority 2018a, 2018b, 2018d).

In the Cape Winelands, with insufficient rehabilitation resources and multiple barriers limiting access to services, most stroke survivors and their caregivers were dependent on CHWs who had no stroke training. In this context, the researcher was approached by a district CBS manager to assist in the development of an appropriate home-based stroke rehabilitation training programme to equip these CHWs to train family caregivers. This community-based programme was developed locally through participatory action research and intersectoral collaboration of service providers, in consultation with stroke survivors and their caregivers, as previously published (Scheffler & Mash 2023). The aim of this study was to evaluate the implementation and immediate effects of the training programme as part of the same participatory action research process.

## Research methods and design

### Study design

This was the last stage of a multistage mixed-method study (Table 1). The first two convergent stages, a quantitative longitudinal survey (Scheffler & Mash 2019) and a qualitative descriptive exploratory study (Scheffler & Mash 2020), were part of the situational analysis informing the planning phase of a professional participatory action research (PAR) study. Professional PAR is defined in the CRASP model as (Hart & Bond 1995; Zuber-Skerrit 1992):

- Critical collaborative inquiry by
- Reflective practitioners being
- Accountable and making the results of their inquiry public
- Self-evaluating their practice and engaged in
- Participative problem solving and continuing professional development.

**TABLE 1:** Overview of the multistage study, procedures and results integrated with the steps of the analyse, design, develop, implement, evaluate model.

Study stage	Steps of the ADDIE model	Procedures		Products
Stage 1	Analyse	Longitudinal survey (Scheffler & Mash 2019)		Demographic and socioeconomic profile, including living conditions and education Function and independence Caregiver strain Patient and caregiver satisfaction with services Acute and primary care services received
Stage 2	Analyse	Qualitative descriptive exploratory study (Scheffler & Mash 2020)		Perceived needs of stroke survivors, caregivers and community health workers
Stage 3	Analyse	Participatory action research using cooperative inquiry	Planning	Design and develop training programme and resources (Scheffler & Mash 2023)
	Design			
	Develop			
	Implement		Action	Pilot training programme and evaluate outcomes
	Evaluate		Observation	
			Reflection	

Note: Please see the full reference list of the article, Scheffler, E. & Mash, R., 2023, ‘Evaluation of a stroke rehabilitation training programme for community-based primary healthcare’, *African Journal of Disability* 12(0), a1137. https://doi.org/10.4102/ajod.v12i0.1137, for more information.

ADDIE, analyse, design, development, implement, evaluate.

This professional PAR study used a cooperative inquiry group (CIG) method (Heron 2014; Higginbottom & Liamputtong 2018; Ramsden et al. 2019). This CIG method has previously been used for both stroke programme and training development (Aslani et al. 2016; Dobe et al. 2022; Krieger et al. 2021). It has also been used in PHC research and programme implementation, as it provides both autonomy to participants and facilitates engagement between community members and respective service providers to address community health problems (Ramsden et al. 2019).

The cyclical steps of planning, action, observation and reflection were aligned with the analyse, design, develop, implement, evaluate (ADDIE) instructional design model (Allen 2006; Mayfield 2011) for development of a training programme (Table 1).

**FIGURE 1:**
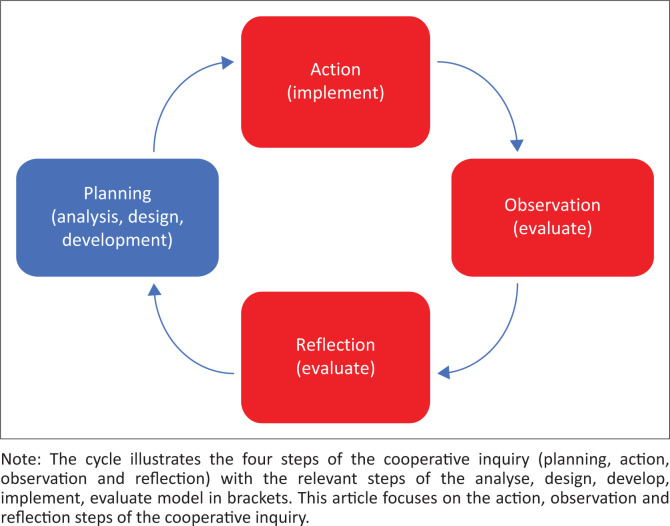
Diagrammatic representation of the methods.

This article focuses on the action, observation and reflection steps of the inquiry, which corresponds with the implementation and evaluation steps of the ADDIE model (Figure 1). Stages 1 and 2 as well as the planning phase of stage 3, detailing the analysis, design and development steps of ADDIE, were previously published (Scheffler & Mash 2019, 2020, 2023).

### Setting

**FIGURE 2:**
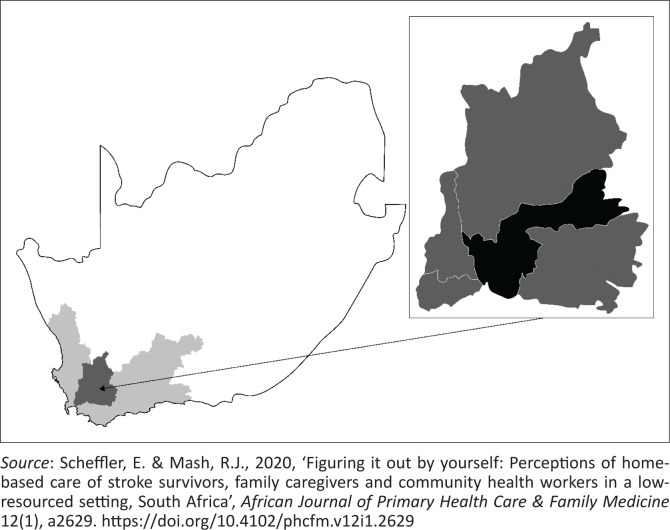
Map of South Africa illustrating the Western Cape province (light grey area), with the Cape Winelands district (darker grey area). The insert shows the Breede Valley subdistrict (black).

The Cape Winelands District in the Western Cape province of South Africa (Figure 2) is a rural agricultural area with small towns, where the majority of the 866 000 population live in poverty and are dependent on state services (Statistics South Africa 2018). The predominant language is Afrikaans, followed by English and isiXhosa. In this low-resourced district with significant health inequities, limited healthcare capacity and funding, limited transport infrastructure and personal and environmental barriers in accessing healthcare services, HCBC is the only resource for many stroke survivors and their caregivers. However, these CHWs have not received any stroke-specific training.

Stroke clinical practice pathways and referral guidelines were absent and stroke care was poorly coordinated (Scheffler & Mash 2019). Stroke survivors were discharged home from acute care to untrained family caregivers after 5 days (Scheffler & Mash 2019). Function and care were limited by numerous environmental barriers such as unavailable or inaccessible services, physical barriers in the home and lack of assistive products (Scheffler & Mash 2019, 2020). Overall, literacy and numeracy of both stroke survivors and caregivers were poor, with more than half having no or only primary school education (Scheffler & Mash 2019, 2020). Knowledge of stroke and recovery, rehabilitation, health services and assistive products was poor.

The study was conducted in the Breede Valley subdistrict (Figure 2) where the CBS management offices are based. Free rehabilitation-related services, delivered by four independent organisations, were available (Table 2). The district health services employed MDPs who previously offered ad hoc training to the CHWs. Boland Hospice, a local non-profit organisation, employed 79 CHWs supervised by 4 nurse coordinators. The Breede Valley Association for the Physical Disabled (APD) offered help with employment and education. Stellenbosch University also placed health science students in the district.

**TABLE 2:** Numbers of the multidisciplinary professionals and community health workers in the Breede Valley subdistrict.

Professionals and health workers	*n*
Physiotherapists	1
Occupational therapists	1
Speech therapists	1
Social workers	1
Oral hygienists	1
Clinical psychologists	1
Dieticians	1
Community health workers	79

### Selection of cooperative inquiry group members

In consultation with the service managers of the five organisations, 27 key role players involved in stroke-related service delivery at PHC level were invited and all but one agreed to participate (Scheffler & Mash 2023). This process is described in detail in a separate article outlining the planning of the inquiry process (Scheffler & Mash 2023). Because of practical considerations, two CIGs were formed. The CBS-CIG consisted of 18 service providers with both clinical and educational expertise and included 5 CHWs, while the MDP-CIG consisted of 8 MDPs (Table 3).

**TABLE 3:** Profile of the two cooperative inquiry groups.

Characteristics	Community-based services CIG			Multidisciplinary professionals CIG	
	Community-based service management	Boland Hospice	Association for physically disabled	District	University
Total participants (*N*)	3	11	4	5	3
**Professions**					
Operational manager	2†	1†	1†	0	0
Nurse (enrolled or professional)	1	4	0	0	0
Social worker	0	1	2	1	0
Occupational therapist	0	0	1	1	1
Physiotherapist	0	0	0	1	1
Oral hygienist	0	0	0	1	0
Speech therapist	0	0	0	1	1
Community health worker	0	5	0	0	0
**Gender**					
Male	0	-	-	1	-
Female	18	-	-	7	-
**Age range (years)**					
20–29	0	-	-	2	-
30–39	7	-	-	2	-
40–49	8	-	-	3	-
50–59	2	-	-	1	-
60+	1	-	-	0	-
**Time in profession (years)**					
1–5	4	-	-	1	-
6–10	5	-	-	4	-
11–20	6	-	-	1	-
21+	3	-	-	2	-

CIG, cooperative inquiry group.

† , Three of the managers were trained healthcare professionals: a social worker, professional nurse and occupational therapist.

### The inquiry process

#### Planning

The first 11 months of planning in 2014–2015 by the two CIGs was described in a separate article (Scheffler & Mash 2023). This included an extensive analysis, which informed the consultative coordinated interprofessional design (Harris et al. 2016) of the training programme (Scheffler & Mash 2019, 2020). This article reports only on the action, observation and reflection steps over 4 months in 2015.

#### Action

The interprofessionally designed training programme (Scheffler & Mash 2023) was delivered to three groups of 24 CHWs, in seven 3-h periods over 10 weeks, according to the training manuals. The trainer’s manual outlined the learning outcomes, content, resources, preparation needed and teaching methods for each session (Table 4). Following adult education principles (Hanger & Wilkinson 2001; Kaufman 2003; Knowles 1970), delivery was interactive, experiential and collaborative, with role-plays and case studies. The curriculum design included principles of a spiral curriculum (Harden & Stamper 1999) where key concepts were revisited to reinforce prior learning and add new depth. Skills learning combined essential theory, modelling and simulated practice with skillful feedback (Hanger & Wilkinson 2001; Kaufman 2003; Maguire & Pitceathly 2002).

**TABLE 4:** Programme outline and time allocation of the home-based stroke training programme.

Session name	Key programmatic outcomes (aims)	Duration in minutes
Introduction	To introduce the trainers and community health workers (CHWs) to each other, and to explain the aims and learning outcomes of the training programme to the CHWs	15
What is a stroke?	To enable CHWs to explain to patients who had a stroke and their family caregivers the causes, symptoms and problems associated with a stroke, as well as recovery after a stroke	55
Stroke services and rehabilitation	To enable CHWs to explain the aims, benefits and principles of stroke rehabilitation to patients who had a stroke and their family caregivers with specific reference to the home-based rehabilitation that patients will receive at primary care level	55
Communication problems	To enable CHWs to guide and support family caregivers to communicate effectively with patients who had a stroke and who experience problems with communication	55
Emotional and social well-being	To establish and maintain healthy supportive relationships between the CHW, patients who had a stroke and their families, and to identify psychosocial risks to the patient’s well-being	180
Problems with the mind and behaviour	To enable CHWs to teach family caregivers to manage and interact effectively with patients who have had a stroke and who are experiencing problems with the mind (cognitive problems) and behaviour	60
Positioning	To enable CHWs to guide and support patients who had a stroke and their family caregivers on how to position and support the patients in bed and in a chair	120
Moving in bed	To enable CHWs to guide and support patients who had a stroke and their family caregivers on how to move the patient in bed and how to help the patient move in bed	180
Transfers	To enable CHWs to guide and support family caregivers on how to transfer or help transfer patients who had a stroke	180
Bladder and bowel management, and using the toilet	To enable CHWs to guide and support family caregivers to improve bladder and bowel management, do safe toilet transfers and select alternative toileting options for the home bedroom	30
Eating, drinking and swallowing safely	To enable CHWs to guide and support family caregivers to help patients who had a stroke and have difficulty eating, drinking and swallowing to do so safely	45
Mouth care	To enable CHWs to teach family caregivers to promote and ensure good mouth and dental care in patients who had a stroke	25
Washing	To enable CHWs to teach family caregivers how to wash a patient who had a stroke and is unable to wash themselves and how to help patients to wash themselves	50
Dressing	To enable the CHW to teach the family caregivers to safely dress the patients who had a stroke and to teach patients how to dress themselves	30
Moving around	To enable CHWs to teach family caregivers how to help patient who had a stroke move around at home in a wheelchair or walking	120
Rehabilitation exercises	To enable CHWs to guide patients who had a stroke and their caregivers, how to do basic rehabilitation exercises	60

**Total time**	**-**	**1260 min (= 21 h)**

CHWs, community health worker.

The researcher and district MDPs from the MDP-CIG delivered the sessions (Table 4) and were assisted by another physiotherapist and physiotherapy assistant with experience in training. The number of trainers per session varied between one and four depending on the content. A trainer to participant ratio of 1:6 was required for learning practical skills, with each trainer supervising two groups.

Training was conducted in the language preferred by the group and was delivered in Afrikaans for two groups and in English for the third group. Everybody was proficient in English reading, writing and speech.

Training was conducted at Boland Hospice during normal working hours. The researcher supplied the training resources such as printed participant and trainer manuals and PowerPoints (which were only available in English for the pilots), exercise mats and equipment.

#### Observation

The CIGs used the following to observe the training programme and its effects:

- Direct observation of all training delivery sessions by CIG members.
- Written and verbal feedback from trainers and CHWs after each session.
- A focus group interview (FGI) with selected CHWs.
- Observation of CHWs working in households by CBS-CIG members.
- Observation of changes in coordination of services by CIG members.

Training attendance ranged from 61 to 72 CHWs per session, with an average attendance of 67 (93%) per session. Reasons for non-attendance included being on leave, missing the transport or prior commitments. Those who missed sessions were required to work through the reference materials and were assisted by the relevant supervisors or MDPs from the CIG groups.

Between one and three CIG members observed each session. Trainers and observers kept written field notes. At the end of each session, the researcher facilitated feedback from the trainers and observers on the session structure, content, instructional strategy and methods, resources, achievement of goals and the trainer’s guide. Observations and comments were recorded using a self-developed structured template.

Community health workers gave written feedback after each session on the achievement of the learning outcomes and their most valuable learning. Before the start of each session, the trainer facilitated feedback from the CHWs on their experiences of implementing skills and knowledge to determine if any changes were required to the previous session. This was audio-recorded and summarised.

Following each session, the researcher, trainers and observers considered the observations from the different sources to reach consensus on what changes were needed. Revisions to the training programme and participant and trainer’s manual were made immediately and reviewed when the session was delivered to the next group.

Following the training programme, a FGI was held with 15 purposively selected CHWs who attended all the training sessions, had worked with stroke survivors and their families during and following the training period, or were part of the CBS-CIG (Table 5).

**TABLE 5:** Profile of the participants in the community health workers’ focus group interview.

Characteristic	*n*	%
**Gender**		
Female	15	-
**Age range**		
20–29	6	40.0
30–39	3	20.0
40–49	4	26.7
50–59	2	13.3
**Time working as CHW (years)**		
< 1	2	13.3
1–5	8	53.3
6–10	5	33.3

CHW, community health worker.

The FGI was led by the researcher using a semi-structured interview guide, which explored their experiences and perceptions of the programme, training resources and how CHWs had applied the content in their work, with specific examples.

The FGI was conducted in Afrikaans, with some English, over 75 min. The audio recording was transcribed verbatim, checked by the researcher and thematically analysed using Atlas-ti^®^ software and the framework method (Mabuza et al. 2014; Pope, Ziebland & Mays 2000). Findings were organised into three main themes related to experiences and perceptions of the training programme; transfer of learning; and professional support (Carter et al. 2014) and reflected on by the CIGs.

#### Reflection

The CIGs met separately to discuss all their observations and reflect on their learning. The meetings were audiotaped and transcribed verbatim. The researcher summarised the consensus of learning in both CIGs. The final integrated consensus of learning and recommendations were validated by both CIGs via email.

#### Quality of the inquiry process

The inquiry process was guided by 10 concepts of quality in cooperative inquiry (Mash & Meulenberg-Buskens 2001). CIG members aligned themselves with the purpose of the research and shared ownership of the inquiry process. The inquiry process was facilitated by the researcher in a consultative and collaborative manner. Being both participants and researchers in the process, CIG members engaged with both delivery and application of the training programme and reflection on their own experiences and observations. All observations, reflections and the emerging consensus of learning were documented. The researcher was responsible for sharing findings and conclusions between the CIGs.

### Ethical considerations

The study was approved by the Health Research Ethics Committee 1 at Stellenbosch University (Ref S13/09/158) and permission was obtained from the Department of Health to conduct the study.

## Results

This section presents the consensus of learning from the two CIGs. In order to illustrate what was learnt and to support its credibility, quotations from the various documents created by the CIGs are added. Personal identifiers are not used in the quotations as they would compromise confidentiality of the participants and therefore codes have been used.

Consensus from the CIGs suggested that the training programme was successful in achieving its objectives. The CIGs noted that out of the 6773 CHW feedback forms received at the end of training sessions, 6764 (99.9%) reported that the learning outcomes were achieved.

### Course structure and training resources

Cooperative inquiry group members found the trainer’s manual to be comprehensive, detailed and user-friendly and felt it made facilitation simple and easy for inexperienced trainers:

‘I think it was comprehensive, and on their [*CHWs*] level, easy to understand and to implement.’ (MDP-CIG)

Community health workers found their manual, particularly the sequential drawings, easy to understand and use, both in the classroom and workplace. Although English was the common language, Afrikaans-speaking CHWs strongly advocated for Afrikaans translations of the manual. Xhosa-speaking CHWs were divided about the need for isiXhosa translations, with the majority favouring English as there is no isiXhosa vocabulary for many of the concepts.

### Training programme design and development

Cooperative inquiry group members found that the training programme promoted a holistic, uniform approach. The modular structure made it easy to deliver the training over a timeframe suitable to the services. Learning was consolidated through a spiral curriculum and by combining theory, modelling and practice with feedback. This meant that the core knowledge and principles were revisited and reinforced through practical activities and case studies:

‘And I think it was nice the way it was designed. The progress, the further we went, the more you see how everything integrates.’ (MDP-CIG)

Despite the step-by-step picture-rich guidelines for practical demonstrations, trainers still modelled the skills in different ways. CIG members suggested to develop video clips to ensure a standardised approach.

Cooperative inquiry group members recognised the importance of analysing learners’ characteristics, learning needs and prior competencies, to ensure appropriate training delivery:

‘Because they, our carers [*CHWs*] are not academics. They are practical people. They are hands on … We’ve learnt that if I implement it, I will remember.’ (CBS-CIG)

Both MDPs and CHWs recognised that rehabilitation was a way of life, rather than a set of exercises:

‘Because it took everyday activities and made it meaningful. It wasn’t just a lot of exercises … to do, it was something meaningful … and practical.’ (MDP-CIG)

‘We know now that every movement you do is exercise. It is an exercise for the patient.’ (CHW-FGI)

One MDP reflected on how this emphasis contributed to refocusing their individual outpatient treatment planning and design, resulting in much more meaningful outcomes than before:

‘I only did sit to stand, something stupid, really very basic. And showed the wife. And he got better quickly, within a week! … It was the fastest I ever made a difference. Because it was something so basic … and targeted. And I think that is good.’ (MDP-CIG)

The therapists reflected on their use of jargon and the importance of using plain language:

‘I realised that sometimes when we talk, we are too clinical. So it is not easy to explain … This is what I struggle with, to break it down to the level that they will understand.’ (MDP-CIG)

### Delivery of training

Training had to consider limited literacy of the CHWs, so trainers read the case studies and instructions aloud. Likewise, the peer assessment checklists were simplified to accommodate slow reading speeds. The interactive delivery was successful in creating a relaxed atmosphere, engaging and motivating the CHWs, who had fun, worked hard and felt safe to ask questions:

‘There was a lot of laughter, a lot of fun. It was not this feeling of [*sigh*] we are in class. You know it was not that heaviness that one gets from the theory sessions.’ (CBS-CIG)

‘It was very good and understandable. It was not boring. You finished up not being tired, because the person who is standing in front of you is explaining and we understand him.’ (CHW-FGI)

Community health workers could identify with the scenarios and characters in the case studies. The case studies required CHWs to role-play both the stroke survivor and caregiver and this experience motivated them to try out the knowledge and skills learnt in the sessions on their own patients:

‘I noticed that as they were talking amongst each other, they were hearing things that they did not know before. But they could immediately click and relate it to their patients. “Okay, so I am going to do this next time I get to A, B or C”.’ (MDP-CIG)

‘It was very well presented. And the case studies worked very well also. Each time, we also were the patient. We enjoyed that a lot.’ (CHW-FGI)

‘The case studies were beautiful. In all the case studies we could see our own patients.’ (CHW-FGI)

Multidisciplinary professionals reflected that they previously perceived CHWs to be uninterested in training. They now realised that several factors contributed to this perception. They had not adequately analysed the CHWs’ learning needs and overestimated their knowledge, resulting in the training being too complex. In addition, they recognised that they had used too much jargon and theory:

‘I think it worked very well, because they had to first problem solve between themselves. “What did she say again about that and so … Oh, yes! Now we can work out the case study. And then we do it practically.” Then it is so much easier, or learning is faster. So, after theory, the case study that had to be completed. And then the questions came up about things they have not fully grasped yet. I think, it really worked well.’ (MDP-CIG)

The methodology was successful in integrating theoretical and practical principles. It made sense to CHWs why things had to be done a certain way:

‘[The training] made things more clear and easier for me. Many of the things we did before. But we did not understand why we were doing it. But we can improve now.’ (CHW-FGI)

Cooperative inquiry group members commented on the studiousness and eagerness with which the CHWs participated and how hard they practiced and worked towards mastering the skills. They recognised the importance of practice with feedback to learn skills:

‘I think everyone was eager to learn, because there was a lot of things they did not know. And I think the practice, the practical sessions, taught them a ***lot***. The fact that you are not only hearing something, but that you are also doing it.’ (CBS-CIG)

The CHWs appreciated that they could practice the skills and get immediate feedback, something they did not often experience in training:

‘Just to sit and listen … No! That is boring! That is where I get lost.’ (CHW-FGI)

‘We practiced! We did not do nothing. There was action! We were doing [things] so that we could learn.’ (CHW-FGI)

The therapists were surprised by the time required for some CHWs to master the skills and knowledge, despite the simplicity of the training programme. They reflected that they previously underestimated how much time was needed for practice:

‘Something that was very valuable to me was that I realized in the sessions that … we assume that they will also know something that is obvious and comes easy to us … but if you actually physically do the practical, then you realize it does not come naturally.’ (MDP-CIG)

The feedback to CHWs from peers and trainers in these sessions was an essential part of the CHWs’ learning. The MDPs found these activities useful in formative assessment of the CHWs. They also suggested that learning could be further strengthened by additional supervised practice, during home visits, as well as formal assessments of knowledge and skills.

### Home- and community-based care service

The training programme defined the CHWs’ roles clearly. This, together with being able to explain their roles, the rehabilitation process and the responsibilities of the caregivers and stroke survivors, helped them to confidently set goals with families and provide motivation:

‘What I observed was that they work much more confidently with the patient. They now know what to do!’ (CBS-CIG)

Where stroke survivors and caregivers were previously passive recipients of the home-based service, CHWs now actively engaged them in a functional, task-oriented approach. Community health workers gave step-by-step instructions, demonstrated, guided and supported the stroke survivors and caregivers in activities such as bed mobility, transfers, washing, dressing and getting out of bed:

‘I was amazed about how confidently the carers [*CHWs*] now interact with the families and train them … No, before, there would have been no training.’ (CBS-CIG)

‘The patient did it [*able to assist*]. She (CHW) encouraged her, and boosted her self-esteem and confidence … And the patient also feels this is about me as patient, I am important.’ (CBS-CIG)

Community health workers felt confident sharing knowledge, teaching skills, providing assistive products and addressing environmental barriers. Their pride and excitement were tangible:

‘Before the patient always struggled [*to transfer*] and two of us had to literally lift him into the wheelchair. We placed the transfer board on the wheelchair. And he transferred easily to the wheelchair. On his own!’ (CHW-FGI)

After having always given the stroke survivor a bed-bath and dressing him, one CHW explained how she patiently guided and motivated him to assist her with these tasks:

‘And he did it! He did it very well! And he was so excited to be able to do it!’ (CHW-FGI)

Another CHW shared how she motivated a stroke survivor who was unsafe because of an unstable ankle to use his ankle-foot orthosis and assisted him with walking. Where before he had to be reminded to use the orthosis, he now asked for it when he got up in the mornings:

‘He feels that the foot splint makes a difference. He says he actually now regrets not wearing it before … It makes such a difference to his walking. He moves around the house so easy now. He couldn’t do it before. Such a difference!’ (CHW-FGI)

Community health workers explained that they now also approached their routine tasks such as giving medication in a more therapeutic way. For example, when supervising and guiding a stroke survivor to take their medication, they encouraged use or support of the affected arm and hand as well:

‘I place the water on the weak side, then I say to her: “You know which hand to use”.’ (CHW-FGI)

Community health workers reported that where they were previously blindsided by poverty and lack of funding in the provision of assistive products, they now had knowledge to make low-cost assistive products such as a self-made plate guards, one-hand spreading boards, commodes and mattress protectors. They enthusiastically shared their experiences and the resulting positive outcomes in empowering caregivers and enhancing dignity and independence in stroke survivors. A CHW shared the outcome of giving advice on basic bladder management and providing a self-made commode:

‘And now it is much better because I don’t get that smell of urine when I enter the house. And it was also not nice for her. But now it is so much better. The smell is hardly there anymore, now that she regularly uses the commode.’

The speech- and language therapist recounted an appropriate referral and management by a CHW who recognised a possible swallowing problem when she observed the stroke survivor choking and coughing when drinking. The CHW also provided appropriate advice and addressed the stroke survivor’s sitting posture and head and neck alignment when drinking.

Not only did the CHWs recognise how their training facilitated independence, but also restored dignity and pride:

‘The training benefitted us a lot, because [*after the stroke*] the patients had lost their pride. They previously did everything for themselves. And since we’ve learnt more about stroke, I think they have now also found themselves again.’ (CHW-FGI)

This resulted in a person-centred approach that was tailored to the needs of individuals and enhancing motivation and dignity.

### Service implications

Community health workers were empowered through this programme to confidently interact with families:

‘Before we did this training, you got to the patient and felt helpless. You did not know what to say. No! But now, you can go with confidence and teach them.’ (CHW-FGI)

Community health workers felt that the training programme defined their roles clearly, and that this, together with being able to explain their roles, the home-based rehabilitation process and the responsibilities of the caregivers and stroke survivors, helped them in goal setting with families and provided motivation:

‘It helps you, otherwise you get burnt out. And tomorrow, you don’t even feel like going there, because you always have to do everything for the person. You must do everything! You must do this, you must put him in bed, you must do that … But now you can explain everything. How it works. It is a change in mindset, but it is better, much better.’ (CHW-FGI)

Cooperative inquiry group members recognised that the training programme was an appropriate community-based intervention that improved PHC service delivery and led to more person-centred care. The CHWs were able to offer a more comprehensive service that improved access to care for stroke survivors. This approach resulted in the appropriate use of human resources on the PHC platform:

‘I think it just enables our people [*CHWs*], they feel more empowered … But is also then impacts that many patients. So, I really pleased that we have been a part of it …We are grateful. And it has identified things like how to work better with the therapists.’ (CBS-CIG)

The MDPs were excited by how the intervention benefitted large numbers of patients and improved both continuity of care and coordination of rehabilitation:

‘I think it [*the training programme*] was very good and the principles were very good. These are the things where they [*CHWs*] can assist us a lot … I think that [*working with CHWs*] would be ideal … I think already our contact sessions with our patients are not enough and so I think we are anyway moving towards that kind of treatment approach that we have our extension which are going to be the home-based carers [*CHWs*].’ (MDP-CIG)

The approach also facilitated interprofessional learning:

‘Before [*the training*], the therapists had absolutely no knowledge about what home-based carers do, but through the training, they got to spend time with them, and they could see what they have learnt and can have more confidence in them, [*and recognise*] that they can be another arm in the field. To fill that role in the community.’ (CBS-CIG)

The training programme promoted a comprehensive, uniform approach to home-based stroke care management in the district. CIG members observed appropriate referrals from CHWs for assessment, intervention and assistive products. They also recognised that the CHWs could provide critical support between discharge from acute care and the first assessment by a therapist. Not only did this strengthen the coordination and continuity of care, but also it extended rehabilitation service into homes of stroke survivors:

‘What also happens with the stroke patients is that when they are discharged from hospital, they – like I had a patient recently who had to wait a month before he could see the therapists. In the meantime, the carer [*CHW*] can fill that gap and the patient will benefit.’ (CBS-CIG)

The MDPs realised that the training programme resulted in an appropriate community-based intervention via CHWs. The MDPs were inspired to use the same principles in the development of training programmes for other chronic conditions as well as in their own practice. However, they recognised their inexperience:

‘We can no longer function in isolation. There are … just far too many people who really need help. And we can’t continue to think, I, myself, cannot produce everything. It cannot continue like this … But to get there, that will require quite a mind shift.’ (MDP-CIG)

Moving away from an individualised therapeutic model left the MDPs conflicted as they experienced it as a loss of a core professional identity. This also created a sense of guilt for not meeting stroke survivors’ expectation themselves:

‘It is quite difficult for me. I think we are very clinical and one-on-one and that is what we do as a [*profession*]. And I think sometimes … I think you kind of feel that your role is being diminished.’ (MDP-CIG)

Current service requirements were designed around individual interventions and this also made it difficult for the MDPs to support CHWs. They pointed out numerous barriers to embedding the intervention in service delivery and the need for a systems approach and strong leadership. They lacked resources, knowledge and skills to develop community-based interventions and training programmes. Another challenge was to improve the referral of clients from acute care to HCBC and MDPs:

‘You know CBS is there. And rehab is there. But you struggle to link these services. You struggle to get a harmonized programme.’ (CBS-CIG)

While multisectoral collaboration, particularly with social workers, was reported as a direct result of the training programme, there was no systematic approach to improve such collaboration. Multidisciplinary professionals also reflected on the shortcomings of their own training to adequately prepare them for PHC and community-based interventions:

‘But since the mind shift is taking place, should this not also be implemented by the universities when they place their students so that the students know that when they come here, that they will not be working clinically, but that they will have a different role?’ (MDP-CIG)

The key learning of the CIGs is summarised in Table 6.

**TABLE 6:** Summary of key learning of the cooperative inquiry groups.

Area of key learning	Learning
Home- and community-based care	The CIGs learnt that the training programme: Defined CHWs’ rehabilitation roles and competencies to fulfil these roles Empowered CHWs to transfer skills, knowledge, and to collaboratively solve problems with confidence Enabled a person-centred approach that tailored training to the needs of individual caregivers and stroke survivors Empowered and motivated caregivers and facilitated independence and dignity in stroke survivors
Training programme design and development	While observing training delivery, the CIGs learnt the importance of: Following a comprehensive multidisciplinary scope, holistic, uniform approach Analysing learners’ characteristics, learning needs, scope of practice and prior competencies Following an appropriate methodology to facilitate interprofessional programme design and development Selecting an appropriate rehabilitation framework to guide the intervention Using plain language Adopting a spiral curriculum that constantly reinforces and expands on training
Delivery of training	While observing training delivery, the CIGs recognised the: Importance of the appropriate instructional strategy and methods to facilitate interactive learning Importance of combining theory, modelling and practice with feedback to learn skills Importance of allowing adequate time to learn practical skills Value of experiential learning that builds on the CHWs’ own experiences Potential value of assessing learning in future by use of more formal assessments and supervision of CHWs in the community
Service implications	Through the design and delivery of the training programme the CIGs: Recognised the appropriateness of a PHC community-based intervention for the setting Learnt how to make use of appropriate use of human resources Learnt how the approaches followed in the design, development and delivery facilitated interprofessional learning Learnt how the process promoted comprehensive, uniform approach Recognised the potential of the programme to strengthen continuity of care Recognised how the programme could extend rehabilitation service into homes Learnt how to achieve therapeutic impact through functional, ‘way of life’ approach Experienced the community-based intervention via CHWs to threaten the loss of core rehabilitation role in MDPs Recognised the need for a systems approach and leadership to embed the community-based intervention in service delivery Recognised challenges in coordinating care across multiple sectors and healthcare levels Recognised the need for PHC training at universities for MDPs

MDP, multidisciplinary professionals; CIG, cooperative inquiry group; PHC, primary healthcare; CHW, community health workers.

## Discussion

This training programme appears to be the first community-based stroke programme specifically designed for a low-resourced PHC setting with delivery by CHWs in the absence of formal rehabilitation services. The positive experiences and observations affirmed the contextual and cultural appropriateness and acceptability of the course structure and content (Knettel et al. 2017), which CHWs found to be relevant, meaningful and easy to understand (Perrin et al. 2010; World Health Organization 2018a).

The interactive training methodology (Abdel-All et al. 2017; Hanger & Wilkinson 2001; Knettel et al. 2017; Knowles 1970; World Health Organization 2018a) and the plain language delivery, as well as image-rich text of the training resources (Doak, Doak & Root 1996) were appropriate to training adults with low literacy levels and resulted in an effective learning environment. Learning was enhanced through the principles of adult education (Kaufman 2003; Knowles 1970), a spiral curriculum (Harden & Stamper 1999) and skills learning (Abdel-All et al. 2017; Kaufman 2003; Knettel et al. 2017; Maguire & Pitceathly 2002) (Kaufman 2003; Maguire & Pitceathly 2002) as has been noted elsewhere (Hanger & Wilkinson 2001; Nadar & McDowd 2008; Pitthayapong et al. 2017; World Health Organization 2018b; Yan et al. 2016b). Community health worker learning can be further strengthened and consolidated by supervised practice in the community (Nesbit & Clark 2019).

The training package followed a functional approach, based on normal movement, focusing on the transfer of practical skills and empowerment of the family caregiver, but at the same time offering a beneficial therapeutic approach to facilitate independence in the stroke survivor. This task-shifting rehabilitation intervention is simple and appropriate to the CHWs’ scope of practice, is in line with the national training of CHWs (South African Qualifications Authority 2018a, 2018b, 2018d), does not overlap with that of therapists or other mid-level community rehabilitation workers and is potentially effective (Zhou et al. 2019).

This approach can contribute to informing a generalist scope of rehabilitation practice and training for CHWs in South Africa. Despite a high burden of impairment and disability, the national CHW training curriculum limits rehabilitation to elective modules (South African Qualifications Authority 2018a, 2018c). It would be better to provide such training as core modules that address the range of impairments and functional problems associated with acute and chronic diseases, disorders, injuries, trauma, disabilities, ageing, congenital and genetic conditions as promoted by the WHO (World Health Organization 2017).

The training programme demonstrated the potential to empower CHWs to define their roles and transfer knowledge and skills to family caregivers. Addressing the stroke survivor’s individual needs appeared to positively impact on their independence and dignity and enhanced a person-centred approach (Bodenheimer et al. 2014a), which reportedly increased stroke survivor and caregiver participation and motivation.

These observations highlighted the feasibility of integrating the programme into PHC to improve continuity of care in the home and coordinate the transition from acute hospital care to HCBC. However, the training programme by itself is not able to ensure change in the design of service delivery. Community-based interventions must be strategically integrated with facility-based services in a collaborative practice (Bodenheimer et al. 2014b; Harris et al. 2016; Kane et al. 2016; Mash et al. 2019). Primary healthcare service barriers such as the lack of clinical practice guidelines, poor coordination and fragmentation of services, and lack of interprofessional and collaborative practice must be addressed (Dookie & Singh 2012; Kringos et al. 2010). Therapists’ roles in PHC must be redefined as they need to be able to design, develop and support community-based PHC interventions and be included in PHC service planning and delivery, with clear roles in supporting CHWs (Dizon et al. 2018; Kane et al. 2016; Naidoo, Van Wyk & Joubert 2016). Therefore, these roles should also be included in undergraduate programmes. The current reengineering of PHC (National Department of Health 2020; ed. Van Rensburg 2012; Western Cape Government 2014) may provide the appropriate backdrop to facilitate these changes.

Although all CIG members were aligned with the research project, high service demands limited ownership and participation in the inquiry (Higginbottom & Liamputtong 2018). This limited their reflection (Higginbottom & Liamputtong 2018) on the design process and service implications, with little emphasis on personal change and learning. Pre- and post-tests and/or summative assessments were not included as part of the initial implementation, but would need to be included in future. This intervention was situated within the context of the local mainstream health system and its allopathic paradigm that did not easily embrace traditional healers or indigenous knowledge on stroke and rehabilitation. The findings of the study can only be transferred to similar contexts and group characteristics.

## Recommendations

The initial evaluation of the programme is positive and congruent with the move towards COPC in South Africa. Further evaluation of both implementation in CBSs and the effectiveness of the intervention are needed. Firstly, the health and psychosocial outcomes should be determined quantitatively. And secondly, implementation research should investigate how to integrate the programme into service delivery, including links to mid-level community rehabilitation workers and therapists. The participatory methodology could provide a foundation for the development of additional community-based interventions. The scope of practice for CHWs should incorporate the roles and competencies defined in the training programme. The training programme could be integrated into pre- and in-service training of CHWs. The district health services should consider adopting the model of CHW-led rehabilitation training of caregivers and the implications for the roles of MDPs in training and support of CHWs.

## Conclusions

Participatory action research provided a useful methodology for an inexperienced PHC interprofessional team to design, develop, implement, evaluate and refine a new home-based stroke training programme. Initial evaluation of the programme was positive in terms of its impact on clarifying the roles of CHWs and empowering them to deliver person-centred services to caregivers and stroke survivors. Implementing community-based programmes requires coordination of care across multiple sectors and healthcare levels and the need for a systems approach and leadership to embed the intervention in service delivery. Further evaluation of the effect on caregivers, stroke survivors and service delivery is needed.
